# SIRT6 Ameliorates Cancer Cachexia–Associated Adipose Wasting by Suppressing TNFR2 Signalling in Mice

**DOI:** 10.1002/jcsm.13734

**Published:** 2025-02-19

**Authors:** Kang Xu, Yida Wang, Fang Wang, Yannan Guo, Yu Ren, Vivien Low, Sungyun Cho, Qingfei Liu, Ying Qiu, Xue Li, Kang Yu, Zhongchi Li, Zhao Wang

**Affiliations:** ^1^ Protein Science Key Laboratory of the Ministry of Education, School of Pharmaceutical Sciences Tsinghua University Beijing China; ^2^ School of Basic Medical Sciences Capital Medical University Beijing China; ^3^ Department of Clinical Nutrition and Department of Health Medicine, Peking Union Medical College Hospital Chinese Academy of Medical Sciences Beijing China; ^4^ Department of Pharmacology Weill Cornell Medicine New York New York USA; ^5^ School of Medicine Tsinghua University Beijing China

**Keywords:** adipose wasting, cachexia, SIRT6, SIRT6 activator, TNFR2 antagonist

## Abstract

**Background:**

Cachexia is a wasting syndrome associated with imbalanced energy metabolism and loss of adipose and muscle tissues and contributes to morbidity and mortality in ageing as well as in patients with severe chronic diseases, including cancer. At present, there are no treatments addressing cachexia that have reached validation to be used in the clinic. In this study, we investigate the protective role of SIRT6, an important regulator of energy homeostasis and health preservation, against Lewis lung carcinoma (LLC)–induced cachexia.

**Methods:**

SIRT6 levels of serum from gastric cancer patients (*n* = 22, 65.27 ± 12.50 years old, 40.9% females) and healthy controls (*n* = 22, 63.50 ± 10.77 years old, 45.4% females) were measured to evaluate the correlation between circulating SIRT6 levels and cancer cachexia development. Ten‐week‐old SIRT6 transgenic (TG) and wild type (WT) male mice injected with LLC cells (1.5 × 10^6^ per mouse) were used to investigate the protective effects of SIRT6 on cachexia‐associated adipose browning and lipolysis and the underlying mechanisms. We explored the effect of SIRT6 on LLC‐conditioned medium induced lipolysis in mature adipocytes, differentiated from primary mouse embryonic fibroblasts (MEFs). We evaluated the in vitro effect of a SIRT6 activator by treatment of MDL800.

**Results:**

SIRT6 concentrations were significantly higher in non‐cachectic cancer patients (3.41 ± 0.30 ng/mL) compared to cachectic cancer patients (3.20 ± 0.23 ng/mL, *p* < 0.01), suggesting the negative correlation between SIRT6 level and cachexia in patients with cancer. SIRT6 overexpression significantly ameliorated tumour‐induced wasting and energy expenditure in white adipose tissues (eWAT mass loss: 66% in WT vs. 32% in TG; iWAT mass loss: 69% in WT vs. 40% in TG) through suppression of browning and lipolysis. In LLC‐induced cachexia, tumour necrosis factor‐α receptor 2 (TNFR2) mediated the inhibition of SIRT6 on lipolytic signalling, because the difference in lipolysis between the WT and SIRT6 knockout group was almost eliminated by TNFR2 neutralizing antibody. Increased serum TNFR2 concentration was found in cachectic cancer patients (690.41 pg/mL in non‐cachectic vs. 1166.98 pg/mL in cachectic patients, *p* < 0.05). A selective SIRT6 pharmaceutical activator, MDL800, could completely reverse LLC‐induced lipolysis in adipocytes.

**Conclusion:**

We found an unexpected beneficial function of SIRT6 in cancer cachexia, demonstrating that increased SIRT6 expression or activity is capable of protecting the host against cachexia‐associated tissue wasting, providing a concept of future therapies for cachexia.

## 1 Introduction

Cachexia is a complex syndrome that arises in various chronic and terminal diseases [1]. Patients with cancer often exhibit signs of varying degrees of cachexia, including a loss of body weight, skeletal muscle mass and white adipose tissue (WAT), leading to physical impairment [2]. In addition, chemotherapeutic drugs, a principal treatment for numerous cancer types, can also contribute to the onset and advancement of cachexia [3]. Cachexia causes frailty in cancer patients and hinders their ability to endure further therapies. Severe cachexia is associated with a poor prognosis and decreased survival, accounting for at least 30% of all cancer deaths [4]. Despite these implications, effective and standard therapeutic interventions to prevent or treat cancer cachexia have yet to be identified.

Although cachexia is often characterized by muscle loss, loss of WAT, which serves as a primary energy storage depot and endocrine organ, typically occurs before the onset of muscle loss during tumour progression [5, 6]. Both epidemiological and clinical research have determined a favourable, life‐extending effect of an above‐normal body weight during end‐stage cancer, a phenomenon termed the ‘obesity paradox’ [7]. Importantly, dysfunction in WAT has been proposed as a diagnostic and prognostic biomarker for cachexia among cancer patients [8]. Previous studies have demonstrated that inhibition of WAT wasting using AMPK‐stabilizing peptide or anti‐PTHrP antibody can ameliorate several symptoms of cancer cachexia [6, 9]. Moreover, overweight and obesity are associated with decreased mortality in the oldest old [10]. Therefore, we hypothesized that maintaining adipocyte homeostasis would hold promise in addressing cachexia.

Sirtuins, including sirtuin 1–7, play a crucial role in regulating different metabolic pathways and have become recognized as important sensors of energy status [11]. SIRT6, an NAD^+^‐dependent histone deacetylase, mono‐ADP‐ribosyltransferase and defatty‐acylase, plays important roles in energy metabolism and lifespan regulation [12]. SIRT6 regulates the activity of key transcription factors and cofactors across various metabolic pathways, connecting nutrients signals to cellular responses to energy requirements [11]. Additionally, SIRT6 overexpression prevents against high‐fat diet (HFD)–induced obesity and related pathological damages [13], while depletion of SIRT6 in brown adipocytes inhibits thermogenesis and leads to obesity [14]. These findings suggest that SIRT6 has protective functions in lipid metabolism. Although cancer cachexia and obesity exist at opposite ends of the energy balance spectrum, many discoveries from recent studies of energy balance dysregulation are relevant to both diseases [15]. In obesity and diabetes animal models, SIRT6 suppresses liver gluconeogenesis genes and ameliorates hyperglycaemia by controlling PGC‐1α [16]. On the contrary, SIRT6 can also activate gluconeogenesis, reversing impaired glucose regulation in old mice livers [17]. Imbalanced operation loops of energy metabolism can lead to either obesity or cachexia; thus, homeostatic regulators of energy metabolism could be effective in the treatment of both conditions. The best documented actions of SIRT6 are on regulation of energy homeostasis, making it a potential therapeutic target for both obesity and cachexia.

In this study, we demonstrate that SIRT6 ameliorates WAT dysfunction and helps prevent cancer‐associated cachexia. We show that circulating SIRT6 levels are negatively associated with cachexia in cancer patients. In addition, SIRT6 overexpression protects mice from cachexia‐related WAT and muscle wasting. SIRT6 decreased aggravated energy expenditure induced by tumour, especially WAT browning and lipolysis. Correspondingly, SIRT6 knockout exacerbated adipocyte lipolysis. Mechanistically, TNFα receptor 2 (TNFR2) mediated inhibition of lipolysis signalling in adipose tissue and cell. Moreover, a pharmacological activator of SIRT6, MDL800, could completely reverse tumour‐induced lipolysis. Our study demonstrates that SIRT6 attenuates energy depletion in WAT and plays a role in the wider context of cancer cachexia. Therefore, enhanced expression or activation of SIRT6 offers potential for mitigating cancer cachexia and enhancing patient survival.

## 2 Materials and Methods

### 2.1 Patients and Sample Collection

Gastric cancer patients (TNM staging III) were recruited between June 2019 and July 2021 at Peking Union Medical College Hospital (PUMCH), Beijing, China (*n* = 22, 13 males and 9 females, 65.27 ± 12.50 years old). This study was approved by the Human Ethics Committee of the PUMCH, and all participants gave written informed consent. The subjects were divided into cancer cachectic patients (*n* = 12), cancer non‐cachectic patients (*n* = 10) and healthy controls (*n* = 22, 12 males and 10 females, 63.50 ± 10.77 years old). Patients were identified as cachectic following the criteria established by the international consensus. Cachexia is diagnosed in individuals who have experienced more than 5% weight loss over the past 6 months, or any level of weight loss exceeding 2% in the same timeframe if accompanied by a BMI of less than 20 kg/m^2^. Healthy controls, with age and gender matching with the cancer group, from the Physical Examination Center of PUMCH were defined as individuals who experienced no noticeable weight fluctuations over the previous year and had a BMI of less than 25 kg/m^2^. In cancer groups (including cancer cachectic patients and cancer non‐cachectic patients), the tumour primary location was stomach. Table S1 displays the general characteristics of cancer patients within each group.

### 2.2 Mouse Experiments

SIRT6 transgenic mice (SIRT6 TG) and SIRT6 knockout mice (SIRT6 KO) were generated in the C57BL/6J mouse strain as previously described [18]. Tail DNA was extracted for genotyping with following PCR primers: forward, 5′‐GCCGTCTGGTCATTGTCAACCTG‐3′; reverse, 5′‐AAAGACCCCTAGGAATGCTCGTCAA‐3′. Positive for 494 bp by PCR production was SIRT6 transgene. SIRT6 TG MEFs were separated from SIRT6 TG embryonic, and WT littermates were used as control. Tail DNA was extracted for genotyping with following PCR primers: forward, 5′‐AGTGAGGGGCTAATGGGAAC‐3′; reverse, 5′‐AACCCACCTCTCTCCCCTAA‐3′. Positive for 453 bp by PCR production was SIRT6 knockout. SIRT6 KO MEFs were separated from SIRT6 KO embryonic, and WT littermates were used as control. Mice were kept in semi‐specific pathogen‐free (SPF) conditions and individually housed throughout the tumour inoculation experiment. They were subjected to a 12‐h light–dark cycle (7 a.m. to 7 p.m.) at a temperature of 23°C and provided with a standard AIN‐93G diet. All animal protocols received approval from the Institutional Animal Care and Use Committee (IACUC) of Tsinghua University.

Lewis lung carcinoma (LLC) cells are known to efficiently form tumours and lead to cachexia; therefore, LLC‐tumour‐bearing mice were widely used as a cancer cachexia murine model. Ten‐week‐old male SIRT6 transgenic mice and littermate wild type mice with C57BL/6J background were injected with 1.5 × 10^6^ LLC cells or 200‐μL PBS into the right flank and then sacrificed on day 21 after tumour injection. Body composition analysis was performed by dual photon absorptiometry (Prodigy, General Electric, USA) before execution. Serum was collected via cardiac puncture before mice were sacrificed. Tumour weight, body weight and weight of different tissues were measured. Tissues for H&E staining were fixed in 4% paraformaldehyde and others were frozen in the refrigerator at −80°C. Non‐tumour side adipose and muscle tissues were collected and analysed.

### 2.3 LLC Culture and Conditioned Medium Preparation

Lewis lung cancer (LLC) cells were cultured in high glucose DMEM (Gibco) supplemented with 10% FBS (Corning) and 1% penicillin/streptomycin (Corning). To prepare LLC‐conditioned‐medium, cells were seeded to achieve a 1:3 confluence. The next day, the culture medium was switched to DMEM (containing 1% penicillin/streptomycin, without FBS) and was collected 24 h later. The medium was centrifuged at 1000 × *g* for 5 min. The supernatant, collected as LLC‐conditioned medium, was then diluted at a ratio of 3:1 with fresh medium for use in stimulating lipolysis [6]. High‐glucose DMEM with no cells kept for 24 h in the incubator was used as control medium [6, 9, 19].

### 2.4 Mouse Embryonic Fibroblasts' (MEFs') Culture and Differentiation

MEFs were isolated from SIRT6 WT, TG and KO embryos on embryonic day 13.5, individually. In brief, the skin tissues, but not head, limbs or other organs, were dissected from embryos. And then, tissues were minced and spread in a culture dish containing high glucose DMEM medium supplemented with 10% FBS and 1% penicillin/streptomycin. After 48 h of cultivation, cells and tissues were digested with 0.25% trypsin/EDTA (Corning) for 2 min at 37°C. Digested cells and tissues were passed through a 70‐μm cell strainer and then centrifuged at 1000 × *g* for 5 min. MEFs pellets were resuspended in complete medium and seeded. MEFs were induced for adipocyte differentiation after reaching 100% confluence. The adipogenic cocktail contains 1‐μM dexamethasone (D4902, Sigma), 5 μg/mL insulin (I9278, Sigma), 0.5‐μM isobutyl methylxanthine (I5879, Sigma) and 1‐μM rosiglitazone (S2556, Selleck) in culture medium [9, 20]. The induction lasted for 4 days, during which the medium was changed once. And then, the medium was changed to maintain medium with 5 μg/mL insulin and 1‐μM rosiglitazone for 48 h. Finally, mature adipocytes were maintained in control high glucose DMEM medium or LLC‐conditioned‐medium supplemented with 10% FBS and 1% penicillin/streptomycin for 24 h. TNFR2 antagonist (anti mouse TNFR2/CD120b/TNFRSR1B Neutralizing Antibody, 50128‐RN204, Sino Biological) (12.5 μg/mL) or 20‐μM MDL800 (SML2925, Sigma) were used to treat cells for 24 h. Medium supernatant and cells were collected for further analysis, individually.

### 2.5 Glycerol Levels Analysis

Glycerol in cell culture supernatant was quantified by EnzyChrom Adipolysis Assay Kit (EAPL003, BioAssay Systems) according to the instructions. Ten‐microlitre sample was used for fluorometric assay at λ_ex_ = 530 nm and λ_em_ = 585 nm by GLOMAX Multi Detection System (Promega). Glycerol concentrations were calculated through the glycerol standard curves and normalized to protein concentrations using the bicinchoninic acid (BCA) assay (PC0020, Solarbio).

### 2.6 cAMP Levels Analysis

cAMP (cyclic AMP) concentrations were quantified by cAMP Assay Kit (ab138880, Abcam) according to the instructions. The fluorescence was measure at λ_ex_ = 540 nm and λ_em_ = 590 nm by GLOMAX Multi Detection System (Promega). cAMP concentrations were calculated through the cAMP standard curves and normalized to protein concentrations using the bicinchoninic acid (BCA) assay (PC0020, Solarbio).

### 2.7 Analysis of Gene Expression (Quantitative Real‐Time PCR)

RNA was extracted from frozen WAT and muscle tissues and cultured cells using Eastep Super Total RNA Extraction Kit (LS1040, Promega) and reverse transcribed using Prime Script RT Mater Mix (RR036Q, Takara). gDNase was used to efficiently remove genomic DNA interference in total RNA. The resultant cDNA was analysed by qRT‐PCR. Primers were purchased from Invitrogen. cDNA (20 ng) and 100 nmol of each primer were mixed with SYBR Green QPCR Master Mix (11171ES03, Yeasen). Gene quantification was performed in triplicate on the CFX96 Real‐Time System (Bio‐Rad). Relative mRNA levels were calculated using the ^ΔΔ^Ct method and normalized to β‐Actin. Primers were used as follows:

*SIRT6*, F: 5′‐CAGTACGTCAGAGACACGGTTG‐3′, R: 5′‐GTCCAGAATGGTGTCTCTCAGC‐3′; *Fbxo32*, F: 5′‐CTTCTCGACTGCCATCCTGGAT‐3′, R: 5′‐TCTTTTGGGCGATGCCACTCAG‐3′; *Mstn*, F: 5′‐AACCTTCCCAGGACCAGGAGAA‐3′, R: 5′‐GGCTTCAAAATCGACCGTGAGG‐3′; *Trim63*, F: 5′‐TACCAAGCCTGTGGTCATCCTG‐3′, R: 5′‐ACGGAAACGACCTCCAGACATG‐3′; *UCP1*, F: 5′‐GCTTTGCCTCACTCAGGATTGG‐3′, R: 5′‐CCAATGAACACTGCCACACCTC‐3′; *PGC1α*, F: 5′‐GAATCAAGCCACTACAGACACCG‐3′, R: 5′‐CATCCCTCTTGAGCCTTTCGTG‐3′; *TNFR1*, F: 5′‐GTGTGGCTGTAAGGAGAACCAG‐3′, R: 5′‐CACACGGTGTTCTGAGTCTCCT‐3′; *TNFR2*, F: 5′‐TGACAGGAAGGCTCAGATGTGC‐3′, R: 5′‐ATGCTTGCCTCACAGTCCGCAC‐3′; *Adiponectin*, F: 5′‐AGATGGCACTCCTGGAGAGAAG‐3′, R: 5′‐ACATAAGCGGCTTCTCCAGGCT‐3′; *ASC1 (Slc7a10)*, F: 5′‐TGCTACGGAGTCACTATCCTGG‐3′, R: 5′‐GCTGAAGACCAGTAGGAATGCC‐3′; *Leptin*, F: 5′‐GCAGTGCCTATCCAGAAAGTCC‐3′, R: 5′‐GGAATGAAGTCCAAGCCAGTGAC‐3′; *β‐Actin*, F: 5′‐CATTGCTGACAGGATGCAGAAGG‐3′, R: 5′‐TGCTGGAAGGTGGACAGTGAGG‐3′.

### 2.8 Western Blot

Proteins were isolated from frozen tissues or adipocytes in culture by lysing in RIPA buffer (Solarbio) supplemented with proteases inhibitor cocktail (B14001, Bimake). The homogenates were centrifuged at 4°C, 12 000 rpm for 15 min, and the supernatants were separated on 12% SDS‐polyacrylamide gels and subsequently transferred onto polyvinylidene fluoride (PVDF) membranes (Millipore). Membranes were hybridized with specific primary antibodies for SIRT6 (ab289970 and ab62739, Abcam), p‐perilipin (p‐PKA Substrate, 100G7E, Cell Signaling Technology), perilipin (ab172907, Abcam), ATGL (ab207799, Abcam; 55190‐1‐AP, Proteintech), p‐HSL (Ser563, 4139S, Cell Signaling Technology), HSL (18381S, Cell Signaling Technology), TNFR1 (3736S, Cell Signaling Technology), TNFR2 (109 322, Abcam), H3K9ac (ab32129, Abcam), H3K56ac (ab238307, Abcam), H3 (ab1791, Abcam) and GAPDH (ab8245, Abcam; AC033, Abclonal). The blots were then incubated with Goat Anti‐rabbit HRP‐conjugated secondary antibody (ab205718, Abcam) or Goat Anti‐mouse HRP‐conjugated secondary antibody (ab205719, Abcam). Protein bound by antibodies was detected using SuperSignal West Pico Chemiluminescent Substrate (Thermo Fisher). Resulting images were captured using Chemi Capture (CLINX), analysed with ImageJ software and normalized.

### 2.9 Haematoxylin–Eosin (H&E) Staining

For H&E staining, all tissues were fixed in 4% paraformaldehyde (Servicebio) at 4°C for 24 h. Samples are embedded in paraffin and then dehydrated. Four‐micrometre sections were stained following the manufacturer's instructions (Servicebio). Images were taken using a bright‐field microscope (Leica). For each section, 3 fields were randomly selected and quantified. Then cross‐sectional areas of myofibers and lipid droplets areas were measured using ImageJ (Fiji). Briefly, the cross‐sectional areas of the myofibers were delineated using the ‘Polygon selections’ tool, followed by area measurement. Lipid droplets were selected by ‘Threshold’ command and then measured. Histopathologic score of muscle sections was determined by both inflammation and degeneration level (3 points each, 6 points in total. 0–3 represents no change, mild change, moderate change or marked change, respectively).

### 2.10 Oil Red O Staining

For Oil Red O staining, cells were washed 2 times with PBS, fixed for 30 min, washed 2 times with ddH_2_O and then immersed in 60% isopropanol for 5 min. Next, cells were stained according to the manufacturer's instructions (Solarbio). Briefly, cells were stained in ORO stain solution for 20 min and then washed 5 times with ddH_2_O. Cells were further stained in Mayer haematoxylin staining solution for 2 min and then washed 5 times with ddH_2_O. Finally, cells were immersed with ORO buffer for 1 min. Photographs were captured using a bright‐field microscope (Leica). Lipid content was determined by extracting the oil red O dye using isopropanol and measuring absorbance of the solution at 510 nm.

### 2.11 Rotarod Testing and Grip Strength Measurement

On the same day prior to euthanizing the mice, the rotarod testing and grip strength of forelimbs were evaluated. The evaluation criterion of rotarod test was the time that mouse spends on the rotating rod before falling (Rotarod test, Unibiolab). The residence times at 12 rpm were recorded. Each mouse was allowed to grab a rod connected to the load cell and at the same time pulled it horizontally away from it with its tail (Grip Strength Meter, Unibiolab). A 30‐s rest was given between each test. The grip strength of each mouse was calculated by averaging the results from five repetitions.

### 2.12 Enzyme‐Linked Immunosorbent Assay (ELISA)

Human serum SIRT6, TNFα and TNFR2 concentrations were analysed by Human NAD‐dependent deacetylase sirtuin‐6 (SIRT6) ELISA kit (CSB‐E17018h, CUSABIO), human tumour necrosis factor α (TNF‐α) ELISA kit (CSB‐E04740h, CUSABIO) and human soluble tumour necrosis factor receptor 2 (sTNF‐R2) ELISA kit (CSB‐E11266h, CUSABIO) according to the manufacturer's instructions, individually. Mouse SIRT6, TNFα and TNFR2 concentrations were analysed by mouse SIRT6 ELISA kit (SEE916Mu, Cloud‐Clone Corp.), mouse tumour necrosis factor α (TNF‐α) ELISA kit (CSB‐E04741m, CUSABIO) and mouse tumour necrosis factor soluble receptor 2 (TNFsR2) ELISA kit (CSB‐E04739m, CUSABIO) according to the manufacturer's instructions, individually. A standard curve was generated by standard protein provided in kit.

### 2.13 Statistical Analysis

All the results were expressed as means ± SEM. Two‐group analysis were performed using two‐tailed Student's *t*‐test. One‐way ANOVA was used for comparison of more than two groups. Two‐way ANOVA was used for multiple comparisons. Data were analysed in GraphPad Prism 9.0 software. *p < 0.05 was considered statistically significant.

## 3 Results

### 3.1 SIRT6 Is Negatively Corelated With Cachexia in Patients With Cancer

**FIGURE 1:**
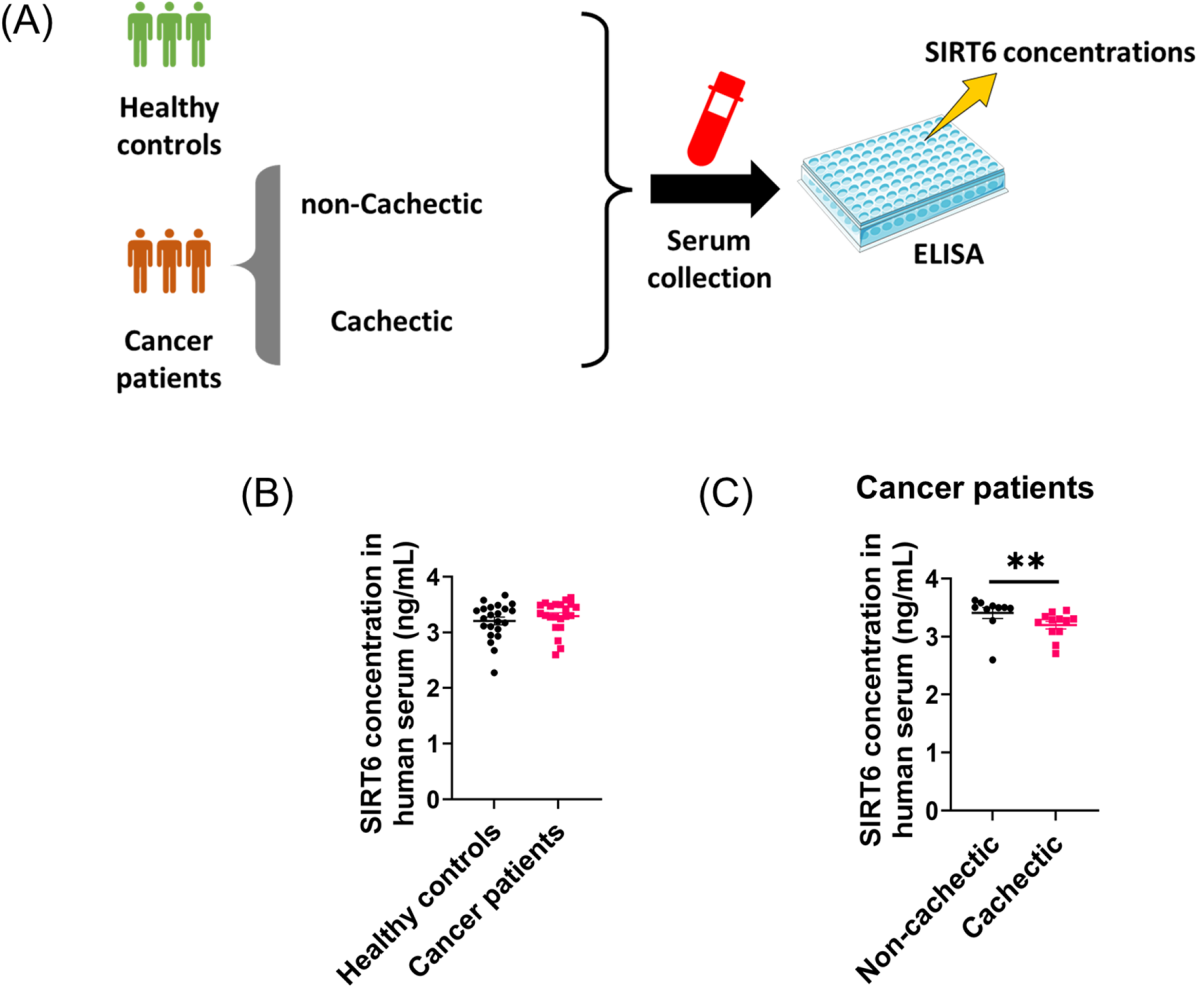
Serum SIRT6 concentrations are associated with cachexia in patients with cancer. (A) Diagram elucidating the collection and analysis of patient serum sample. Serum collected from healthy controls, cachectic cancer patients and non‐cachectic cancer patients, were used to measure SIRT6 concentrations by enzyme‐linked immunosorbent assay (ELISA). (B) Serum SIRT6 concentrations of healthy controls (*n* = 22) and cancer patients (*n* = 22) were compared. (C) Serum SIRT6 concentrations of cachectic cancer patients (*n* = 12) and non‐cachectic cancer patients (*n* = 10) were compared. **p < 0.01.

To examine whether SIRT6 levels are related to the extent of cachexia, we measured SIRT6 levels in a cohort of patients with gastric cancer (TNM staging III). For this analysis, we divided the cancer patients into two groups (cachectic or non‐cachectic) according to the clinical diagnostic criteria of cancer cachexia [21] and used healthy volunteers as the controls (Figure 1A). Table S1 provides a summary of the baseline characteristics of the cancer patients.

Recently, serum SIRT6 has been used to assess health or disease state. For instance, serum SIRT6 levels decline with age in healthy individuals, and this change may underlie many of the metabolic shifts that occur with ageing [22]. In addition, in patients with stable angina or acute coronary, decreased serum SIRT6 expression is observed [23]. We measured the serum SIRT6 levels in cancer patients and age‐matched control subjects and found that while there was no difference between healthy and cancer patients (Figure 1B), SIRT6 concentrations were significantly higher in non‐cachectic cancer patients compared to those with cachexia (Figure 1C). This shows that the circulating SIRT6 level is negatively correlated with cachexia development in cancer patients and suggests that SIRT6 may have a protective effect against cancer cachexia.

### 3.2 SIRT6 Overexpressed Mice Are Resistant to Tumour‐Driven Cachexia

To investigate whether systemic SIRT6 levels play a protective role against cancer cachexia, whole‐body SIRT6 transgenic (SIRT6 TG) mice were used. mRNA levels of SIRT6 were increased 4–7 folds in the adipose and 2–6 folds in muscle tissues (Figure S1A,B), while protein levels were increased 2–3 folds (Figure S1C,D). Serum SIRT6 concentration was also significantly increased in SIRT6 TG mice (682.07 ± 128.33 pg/mL) compared with WT mice (437.74 ± 97.14 pg/mL) (Figure S1E).

**FIGURE 2:**
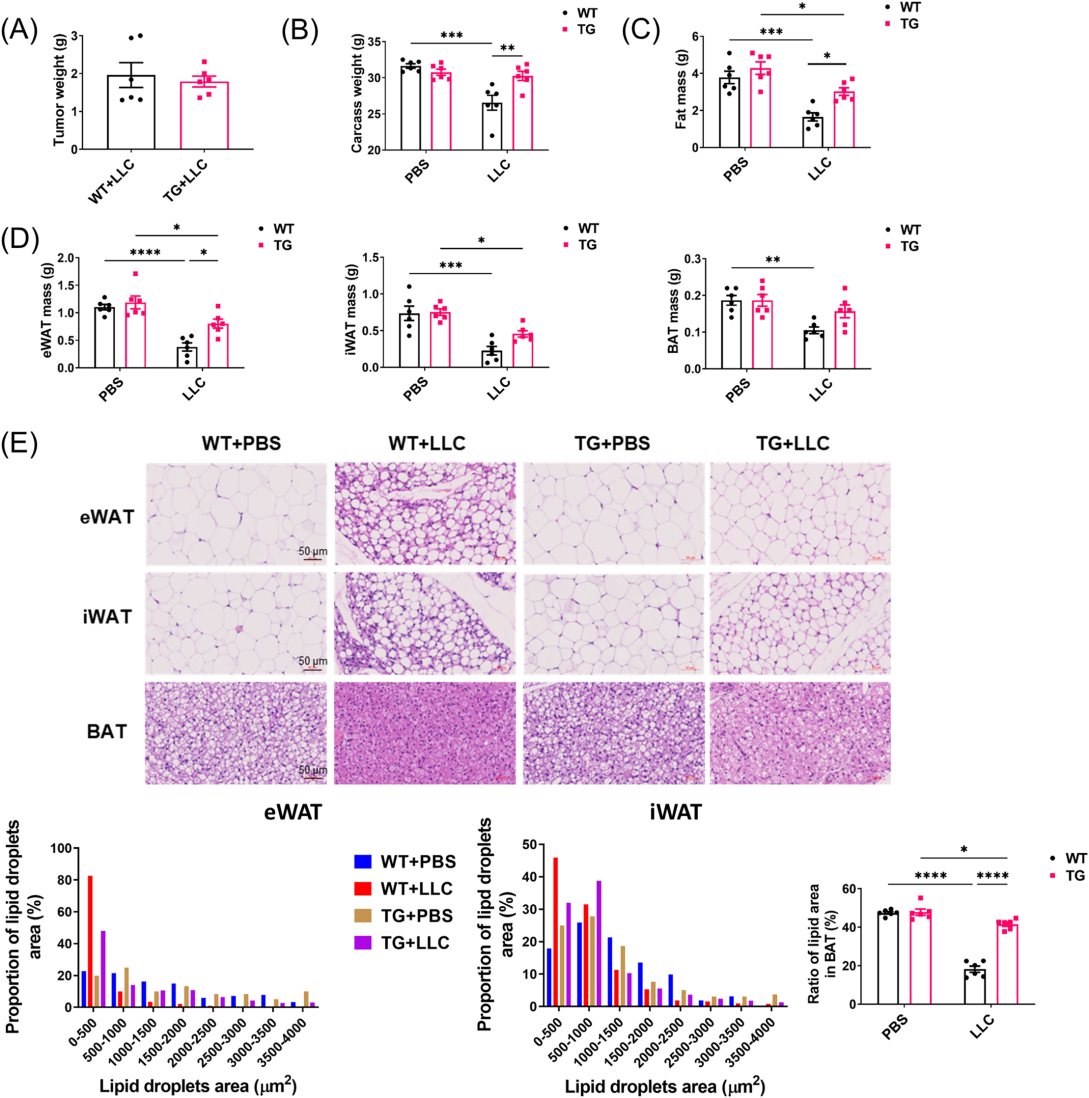
SIRT6 overexpression prevents body weight loss and adipose tissue wasting in tumour‐bearing mice. (A–D) SIRT6 transgenic (TG) and wild type (WT) mice were inoculated with LLC cells or PBS and euthanized 21 days after tumour injection. Tumour weight (A), carcass weight (B), weight of total fat tissue (C), weight of epididymal white adipose tissue (eWAT), inguinal white adipose tissue (iWAT) and brown adipose tissue (BAT) (D) were measured (*n* = 6 per group). (E) Representative H*&*E‐stained images of eWAT, iWAT and BAT. Adipocytes size distribution in eWAT and iWAT and ratio of lipid area in BAT were quantified (*n* = 6 per group). *p < 0.05, **p < 0.01, ***p < 0.001, ****p < 0.0001.

To directly analyse the effect of SIRT6 on the alleviation of cancer cachexia, SIRT6 TG mice and wild type (WT) littermates were subcutaneously injected with Lewis lung carcinoma (LLC) cells and measured after 3 weeks. Tumour weights were similar between WT + LLC and TG + LLC mice, suggesting that the SIRT6 does not affect tumour growth in mice (Figure 2A). However, WT + LLC mice displayed significant carcass weight (determined by subtracting tumour weight from total body weight) loss over 3 weeks, while there was no significant reduction in the weights of TG + LLC mice (Figure 2B). SIRT6 overexpression attenuated adipose tissue wasting (Figure 2C) in epididymal white adipose tissues (eWAT), visceral white fat depots and inguinal white adipose tissues (iWAT), a form of subcutaneous fat and interscapular brown adipose tissue (BAT) (mass loss: 66% in WT vs. 32% in TG of eWAT, 69% in WT vs. 40% in TG of iWAT and 44% in WT vs. 16% in TG of BAT) (Figure 2D). The protective effect of SIRT6 was particularly pronounced in eWAT, as there was a significant difference between WT + LLC and TG + LLC groups (Figure 2D). We observed reduced lipid droplet area in both eWAT and iWAT in WT + LLC mice compared to TG + LLC mice, and decreased BAT lipid accumulation in WT + LLC, but not TG + LLC mice (Figure 2E).

We next examined the influence of SIRT6 on muscle atrophy and found that SIRT6 overexpression also prevented muscle loss by LLC xenografts to a certain degree (Figure S2A, mass loss of gastrocnemius muscle: 18% in WT + LLC mice vs. 11% in TG + LLC mice). The retention of muscle mass was reflected in improved muscle function, as shown by longer residence time in the rotarod test and increased grip strength (Figure S2B,C). Decreased physical activity also demonstrated the frailty of WT + LLC mice compared to TG + LLC mice. Gastrocnemius muscle sections were stained with H&E to examine muscle fibre architecture and cross‐sectional area, which demonstrated larger muscle fibre size and lower levels of inflammatory infiltration in TG + LLC mice compared to WT + LLC mice (Figure S2D). SIRT6 overexpression also reduced the expression of atrophy‐associated genes in skeletal muscle, including *Fbxo32*, *Mstn* and *Trim63* (encoding for atrogin 1, myostatin and MuRF1, respectively) (Figure S2E). Together, our data support a protective role of SIRT6 overexpression against tumour‐driven cachexia, including amelioration of adipose tissue wasting and muscle atrophy.

### 3.3 SIRT6 Decreases Adipose Tissue Browning and Lipolysis Induced by Tumour in Mice

**FIGURE 3:**
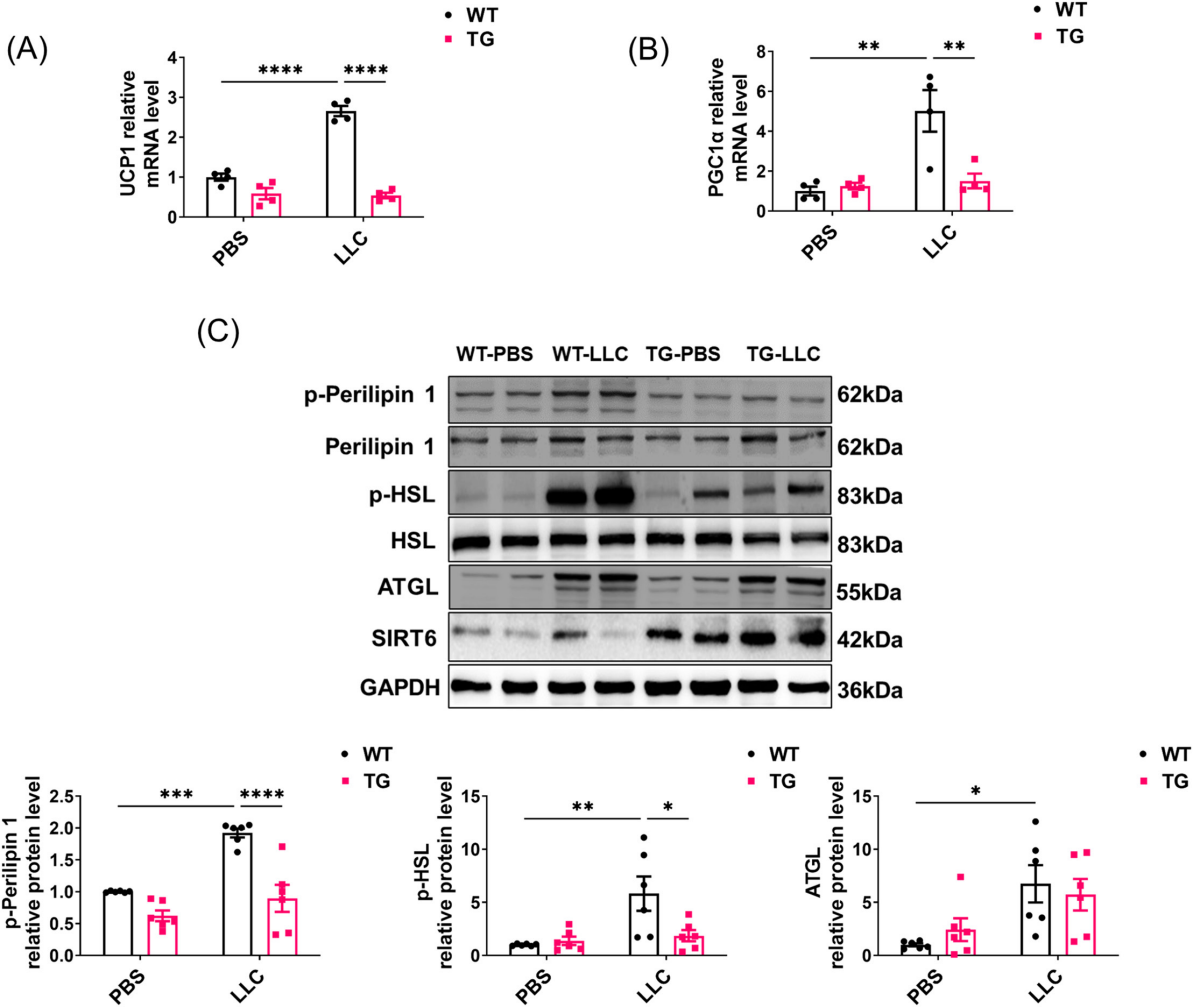
SIRT6 overexpression decreased the expression of genes involved of browning and lipolysis. (A) The mRNA levels of UCP1 in the eWAT were determined by qRT‐PCR (*n* = 4 per group). (B) The mRNA levels of PGC1α in the eWAT were determined by qRT‐PCR (*n* = 4 per group). (C) The expression and phosphorylation levels of lipolysis associated proteins in the eWAT from WT + PBS, WT + LLC, TG + PBS and TG + LLC mice were determined by western blot (*n* = 6 per group). *p < 0.05, **p < 0.01, ***p < 0.001, ****p < 0.0001.

To analyse how the tumour affects adipose tissue in WT and TG mice on a molecular level, we focused on eWAT tissue, which was better improved than iWAT by SIRT6 overexpression (Figure 2D). eWAT has been the focus for cachexia‐associated white adipose browning and lipolysis [24, 25]. In response to tumour growth, eWAT exhibited higher expression of *Ucp1* and *Ppargc1a*, encoding UCP1 and PGC1α, respectively, which are involved in the adipocyte metabolic switch to a thermogenic fat‐burning state termed ‘browning’. Correspondingly, SIRT6 overexpression significantly prevented the increased expression of both genes (Figure 3A,B). These data were consistent with the histological results shown in Figure 2E. Adipocyte lipolysis has been reported to be elevated due to elevated expression of lipolytic enzyme ATGL, along with phosphorylation of HSL and Perilipin 1 [26]. Accordingly, our western blot results showed higher expression of ATGL as well as increased phosphorylation of perilipin 1 and HSL in WT + LLC mice compared with TG + LLC mice (Figures 3C and S3). This was consistent with that the lipid droplets were smaller in WAT of WT + LLC mice compared with TG + LLC mice (Figure 2E), illustrating that SIRT6 overexpression can decrease cancer‐induced WAT browning and lipolysis in vivo.

### 3.4 SIRT6 Attenuates Lipolysis Induced by LLC Cell‐Conditioned Medium in Mature Adipocytes

**FIGURE 4:**
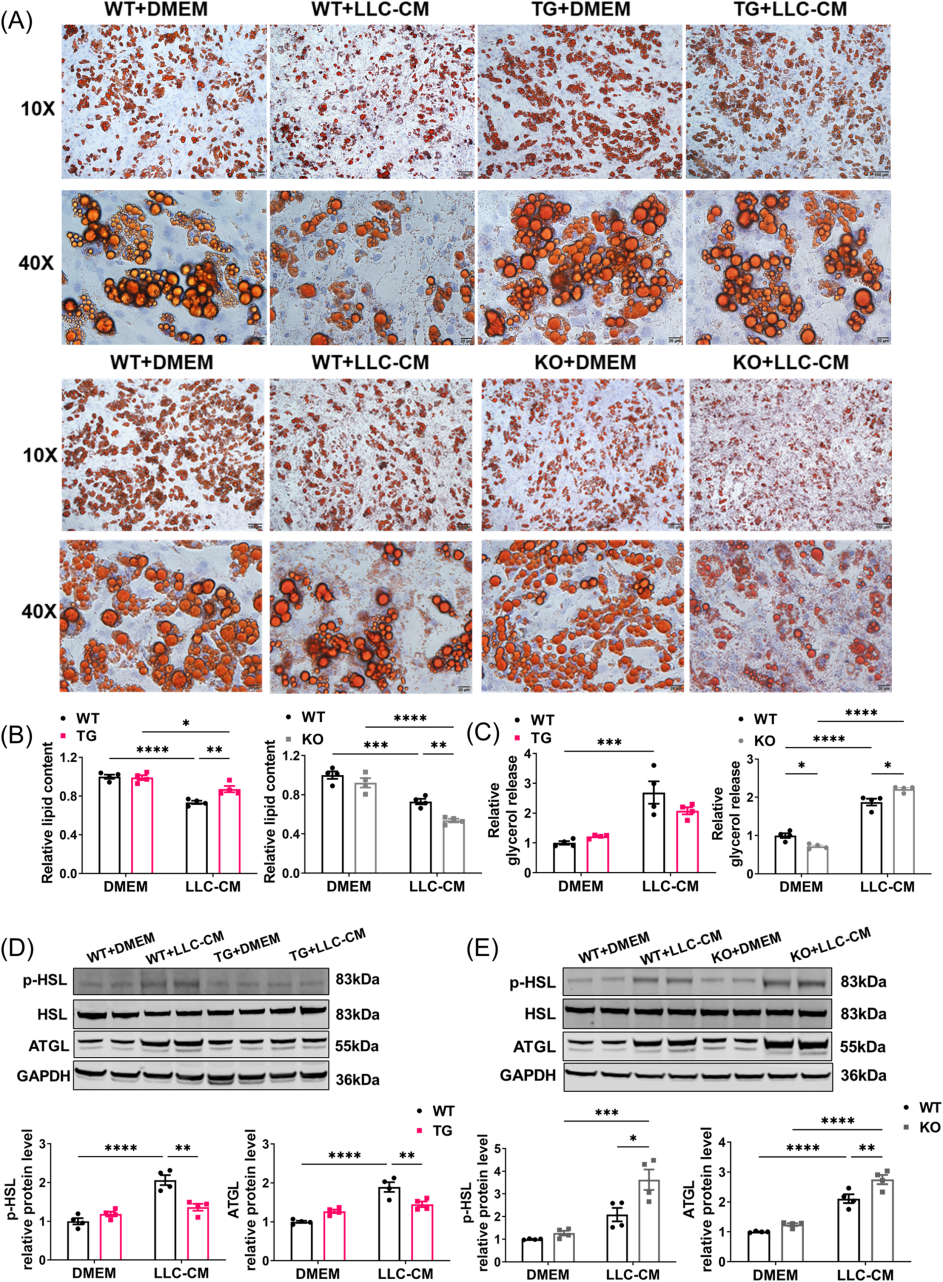
The effect of SIRT6 overexpression and knockout on adipocytes lipolysis. (A) Mouse embryonic fibroblasts (MEFs) isolated from SIRT6 transgenic (TG) and wild type (WT) embryos were induced differentiation into mature adipocytes. Adipocytes were treated for 24 h with LLC cell‐conditioned medium (LLC‐CM) or control medium (DMEM). Representative images of oil red O staining were shown. Mouse embryonic fibroblasts (MEFs) isolated from SIRT6 knockout (KO) and wild type (WT) embryos were induced differentiation into mature adipocytes. Adipocytes were treated for 24 h with LLC cell‐conditioned medium (LLC‐CM) or control medium (DMEM). Representative images of oil red O staining were shown. (B) The quantification of lipid content was shown (*n* = 4 per group). (C) Relative glycerol release in medium supernatant were measured (*n* = 4 per group). (D) The expression of ATGL and phosphorylation levels of HSL in different adipocytes were determined by western blot (*n* = 4 per group). *p < 0.05, **p < 0.01, ***p < 0.001, ****p < 0.0001.

To further validate the protective effects of SIRT6 on tumour‐induced lipolysis, we first derived mouse embryonic fibroblasts (MEFs) from SIRT6 KO, SIRT6 WT or SIRT6 TG mice and differentiated them into mature adipocytes (verified by significantly increased expression of mature adipocyte markers including adiponectin, ASC1 and leptin, Figure S4A) [27], before treating them with LLC cell‐conditioned medium (LLC‐CM). The overexpression of SIRT6 in SIRT6 TG adipocytes and deficiency of SIRT6 in SIRT6 KO adipocytes were verified individually (Figure S4B,C). Upon treatment, we observed reduced lipid droplet size and lipid content with oil red O staining, suggesting increased lipolysis in WT + LLC‐CM adipocytes compared to TG + LLC‐CM adipocytes (Figure 4A,B). In line with this, SIRT6 knockout aggravated the adipose lipolysis induced by LLC cell‐conditioned medium, indicated by the smaller droplet size and lower lipid content in KO + LLC‐CM adipocytes than those in WT + LLC‐CM adipocytes (Figure 4A,B). The release of glycerol into the medium serves as an indicator of triglyceride lipolysis in adipocytes [28]. The results of relative glycerol release into the medium after a 24‐h stimulation corroborated the results of oil red O staining (Figure 4C). Moreover, we found the significantly increased expression of ATGL and phosphorylation of HSL in WT+LLC‐CM adipocytes, but not in TG+LLC‐CM adipocytes. As expected, the increase in expression of ATGL and phosphorylation of HSL were further enhanced by SIRT6 deficiency (Figures 4D,E and S4D,E). Our findings show that SIRT6 overexpression attenuates, but SIRT6 knockout exacerbates, adipocyte lipolysis induced by LLC cell‐conditioned medium in cell, further validating the beneficial effects of SIRT6 against lipolysis in cancer cachexia‐associated adipocytes.

### 3.5 TNFR2 Mediates the Inhibition of SIRT6 on LLC‐Induced Lipolysis

**FIGURE 5:**
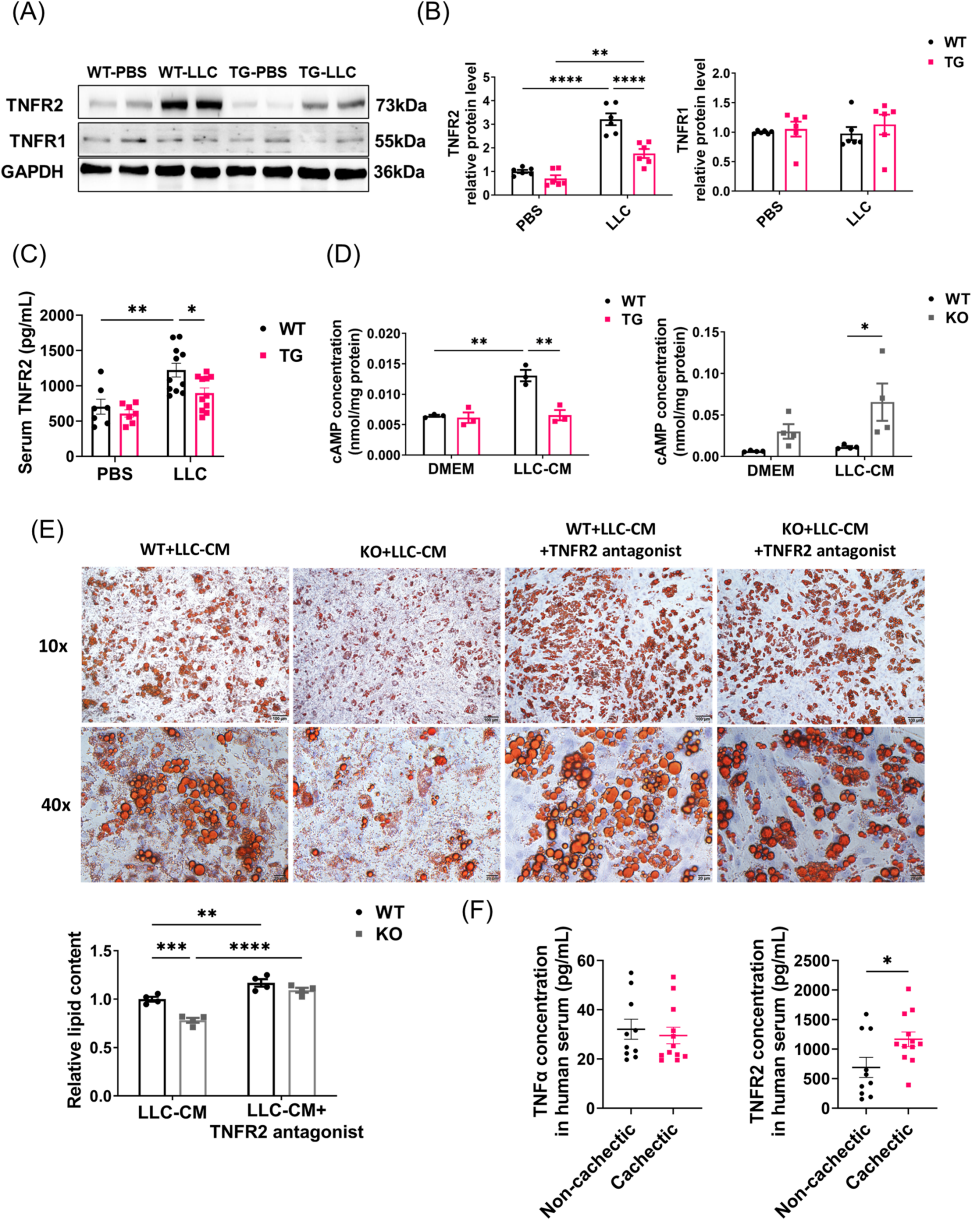
SIRT6 functions in lipolysis mediated by TNFR2. (A–B) Tumour necrosis factor receptor (TNFR1 and TNFR2) protein levels in the eWAT from WT + PBS, WT + LLC, SIRT6 TG + PBS and SIRT6 TG + LLC mice were determined by western blot (*n* = 6 per group). (C) Serum TNFR2 concentrations in mice were measured by ELISA (PBS, *n* = 7 per group; LLC, *n* = 11 per group). (D) cAMP levels in WT + DMEM, WT + LLC‐CM, TG + DMEM, TG + LLC‐CM adipocytes (*n* = 3 per group) or WT + DMEM, WT + LLC‐CM, KO + DMEM, KO + LLC‐CM adipocytes (*n* = 4 per group) were measured. WT + DMEM (WT adipocytes were treated with DMEM medium), WT + LLC‐CM (WT adipocytes were treated with LLC cell‐conditioned medium), TG + DMEM (SIRT6 TG adipocytes were treated with DMEM medium), TG + LLC‐CM (SIRT6 TG adipocytes were treated with LLC cell‐conditioned medium), KO + DMEM (SIRT6 KO adipocytes were treated with DMEM medium) and KO + LLC‐CM (SIRT6 KO adipocytes were treated with LLC cell‐conditioned medium). (E) MEFs isolated from KO and WT embryos were induced differentiation into mature adipocytes. Adipocytes were treated for 24 h with DMEM, LLC cell‐conditioned medium and a combination of LLC cell‐conditioned medium and 12.5 μg/mL TNFR2 antagonist. Representative images of oil red O staining and the quantification of lipid content were shown (*n* = 4 per group). (F) Serum TNFα and TNFR2 concentrations of cachectic cancer patients (*n* = 12) and non‐cachectic cancer patients (*n* = 10) were compared. *p < 0.05, **p < 0.01, ***p < 0.001, ****p < 0.0001.

We explored the molecular mechanism underlying SIRT6 suppression of lipolysis in adipose tissues. It has been reported that malignant tumours release cytokines into the circulation, especially TNFα, which can induce cachexia systemically [26]. TNFα can act upon white adipose tissue by interacting with specific membrane surface receptors to exert downstream effects including the dysregulation of lipid metabolism. Expression levels of TNFR2 (TNFα receptor 2), but not TNFR1 (TNFα receptor 1), was significantly increased in WT + LLC mice compared with TG + LLC mice (Figure 5A,B). In line with this, we observed increased serum TNFR2 levels with LLC implantation, while SIRT6 overexpression significantly reduced this effect (Figure 5C). We also observed decreased TNFR2 expression in SIRT6 TG adipocytes under LLC‐conditioned medium and increased TNFR2 expression in SIRT6 KO adipocytes compared with that in WT groups (Figure S5A,B). By contrast, we did not observe consistent differences in expression of TNFR1 among WT, TG and KO adipocytes (Figure S5C,D).

Considerable amount of TNFα protein (100–150 pg/mL on average) was detected in LLC‐conditional medium (Figure S5E). Activation of the TNFα receptor leads to the phosphorylation of perilipin 1, likely through elevated cAMP levels that subsequently activate PKA [26]. We observed lower cAMP concentration in TG + LLC‐CM adipocytes compared to WT + LLC‐CM adipocytes and higher cAMP concentrations in KO + LLC‐CM adipocytes than in all other groups (Figure 5D). To validate the contribution of TNFR2 to LLC‐induced lipolysis, WT + LLC‐CM and KO + LLC‐CM adipocytes were treated with a TNFR2 antagonist (TNFR2 Neutralizing Antibody), which significantly attenuated the lipolysis in both groups (Figure 5E). The difference in lipolysis between the WT + LLC‐CM and KO + LLC‐CM group was almost eliminated by TNFR2 inhibition, as measured by oil red O staining and lipid content quantification (Figure 5E), suggesting that inhibition of the TNFα‐TNFR2 interaction can efficiently mimic the effect of SIRT6 on lipolysis. Moreover, we measured the TNFR2 levels in muscle tissues of SIRT6 TG mice and WT mice, with or without tumour. We also observed an impressive increase of TNFR2 expression under tumour‐bearing condition and found that overexpression of SIRT6 led to a reduction in TNFR2 mRNA levels (Figure S5F).

To further validate the correlation between elevated TNFR2 levels and increased severity of cachexia, we measured TNFα and TNFR2 concentration in the serum of cancer patients. There was no difference of TNFα concentration between non‐cachectic and cachectic cancer patients (Figure 5F). However, increased serum TNFR2 concentration was found in cachectic cancer patients compared with non‐cachectic cancer patients (Figure 5F). Thus, our data suggest that SIRT6 attenuation of cachexia‐associated adipose lipolysis through the suppression of TNFR2.

### 3.6 Pharmacological Activation of SIRT6 Reverses Tumour‐Induced Lipolysis

**FIGURE 6:**
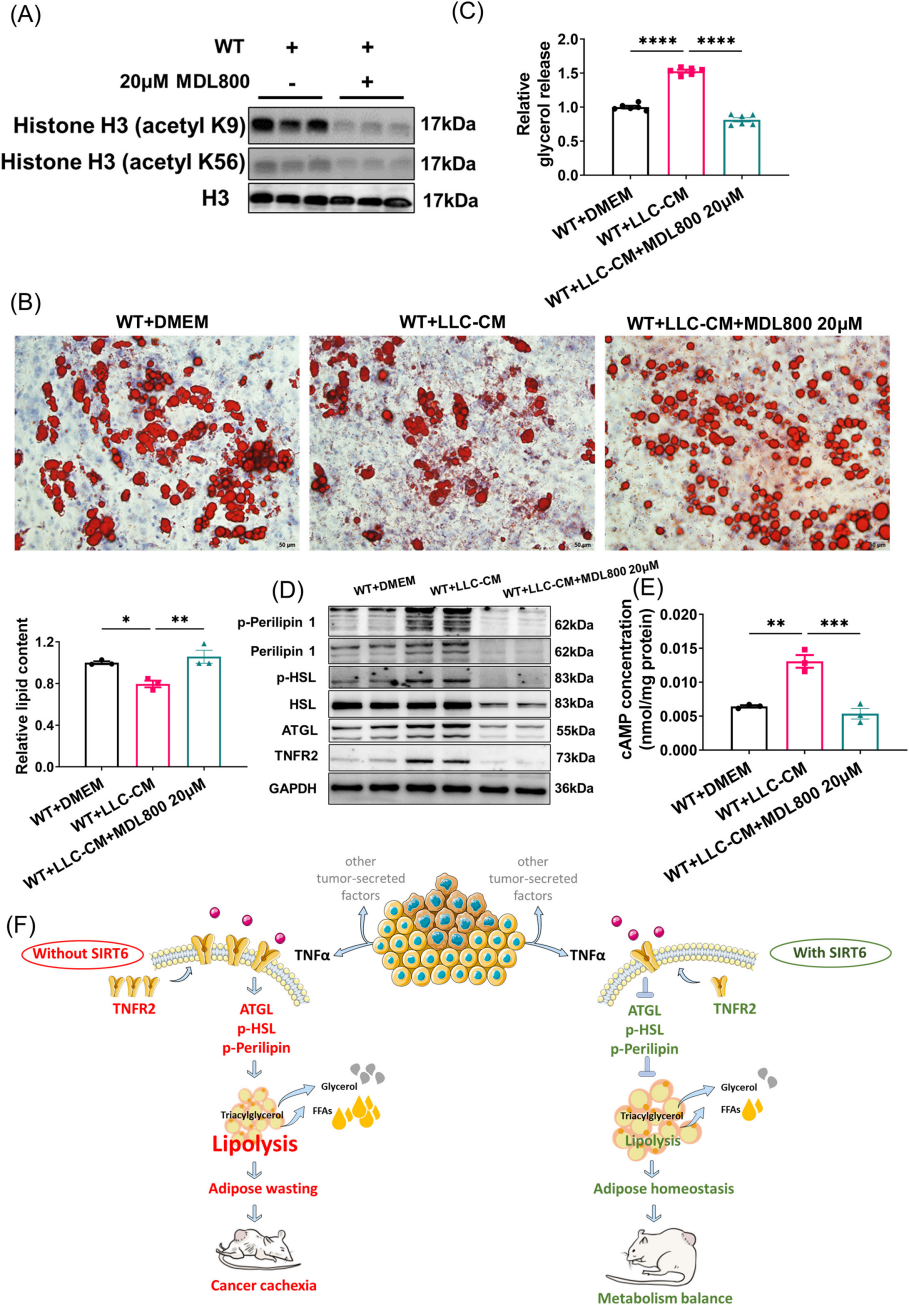
MDL800 reverses LLC‐induced adipocytes lipolysis. (A) Western blot analysis of SIRT6 and its substrates H3K9ac and H3K56ac in WT adipocytes with or without 20‐μM MDL‐800 treatment for 24 h (*n* = 3 per group). (B) MEFs isolated from WT embryos were induced differentiation into mature adipocytes. Adipocytes were treated for 24 h with DMEM, LLC cell‐conditioned medium and a combination of LLC cell‐conditioned medium and 20‐μM MDL800. Representative images of oil red O staining and the quantification of lipid content were shown (*n* = 3 per group). (C) Glycerol levels in medium supernatant were measured (*n* = 6 per group). (D) The expression and phosphorylation levels of lipolysis associated proteins in the WT + DMEM, WT + LLC‐CM and WT + LLC‐CM + 20‐μM MDL800 were determined by western blot (*n* = 4–6 per group). (E) cAMP levels in WT + DMEM, WT + LLC‐CM and WT + LLC‐CM + 20 μM MDL800 adipocytes were measured (*n* = 3 per group). (F) Role of SIRT6 in cancer cachexia. TNFα, secreted from tumour cell, interacts with membrane TNFR2 and then induces the activation of lipolysis signalling pathway, mainly indicated by increased expression of ATGL and over‐phosphorylation of HSL and Perilipin 1. Without SIRT6, lipolysis is enhanced due to increased expression of TNFR2 and interaction of TNFα and TNFR2, which led to adipose wasting in tumour‐bearing mice. When SIRT6 is overexpressed or pharmaceutically activated, decreased expression of TNFR2 attenuates the activation of lipolysis signalling, which led to adipose homeostasis and then metabolic balance in mice. *p < 0.05, **p < 0.01, ***p < 0.001, ****p < 0.0001.

To determine if pharmacological activation of SIRT6 would similarly inhibit cachexia‐associated adipose lipolysis, we used MDL800, a selective agonist of SIRT6, to treat adipocytes [29]. In line with results of this report, MDL800 decreased H3K9ac and H3K56ac levels in WT adipocytes (Figures 6A and S6A). MDL800 (20 μM) completely reversed lipolysis induced by LLC conditioned medium‐induced, as measured by oil red O staining, lipid content quantification and glycerol release analysis (Figure 6B,C). Additionally, MDL800 abolished phosphorylation of perilipin 1 and HSL, expression of ATGL and cAMP production, demonstrating the complete inhibition of tumour‐induced lipolysis (Figures 6D,E and S6B). Correspondingly, increased TNFR2 expression upon culture with LLC‐conditioned medium was also blocked by MDL800 (Figures 6D and S6B). In conclusion, pharmacological activation of SIRT6 can effectively reverse cancer cachexia‐related lipolysis, presenting a promising mechanism and drug candidate to reduce cancer‐associated mortality.

## 4 Discussion

Cachexia is a condition marked by dysregulated energy balance, systemic inflammation and atrophy of WAT and skeletal muscle. Due to the multifactorial nature of cachexia and the tendency to prioritize tumour cell elimination in treatment, there are limited therapeutic options addressing cachexia. While nutritional supplementation is often used in efforts to attenuate cachexia‐related phenotypes, the beneficial effect is often minimal, pointing to a systemic homeostatic dysregulation at the root of the problem. Thus, cachexia remains a critical challenge in the field of cancer treatment. Previous reports have demonstrated that SIRT6 functions in the maintenance of energy metabolism and general health. In vivo, SIRT6 overexpression offers enhanced defence against diet‐induced metabolic challenges in mice, with SIRT6 transgenic male mice displaying longer lifespans than their wild type peers [13, 17]. In our previous work, we have demonstrated that SIRT6 overexpression improves resistance to acute kidney injury from cisplatin in mice [18]. Recently, we have also identified a protective role of SIRT6 in doxorubicin‐induced cardiac dysfunction [30]. Cisplatin and doxorubicin are FDA‐approved chemotherapeutic drugs, and we have found a protective benefit of SIRT6 against side effects of commonly used cancer treatments. In this study, we identify a novel role for SIRT6 in the treatment of cancer, describing its benefit in ameliorating cancer‐induced cachexia and providing further rationale for investigation of SIRT6 activators in cancer treatment protocols (Figure 6F).

SIRT6 is widely expressed in the body, and plays different protective roles in various tissues and organs. As cachexia is a multi‐organ syndrome involving not only the adipose tissue but also the brain, liver, pancreas, gut and muscle [31], we used a globally overexpressing SIRT6 mouse model rather than adipose tissue‐specific overexpression model. Ubiquitous overexpression of SIRT6 can effectively attenuate the tumour‐induced lipolysis in adipose tissue, further in vitro experiments suggest SIRT6 can directly prevent tumour‐induced lipolysis in adipocyte. SIRT6 has been reported to block the cachexia‐associated muscle atrophy by regulating multiple targets [32]; however, our study proposes a novel mechanism of SIRT6 improving adipose metabolism, undiscovered in muscle, suggesting that SIRT6 functions in different tissues through different mechanisms. As for whether the protective effect of SIRT6 comes from other tissues or organs remains to be clarified.

We also identified a novel function of TNFR2 in cachexia. TNFα activity is mediated through two structurally distinct receptors, TNFR1 and TNFR2. It was previously believed that signalling through TNFR1 was the primary contributor to cachexia‐associated WAT wasting [33]. However, knockout of TNFR1 in mice was unable to fully prevent cachexia [34], leading us to reconsider the involvement of TNFR2. We observed that under tumour stimulation, there was a notable rise in TNFR2 expression, but not TNFR1, in both WAT and adipocytes. TNFR2 is a cell‐surface receptor that plays a crucial role in cell survival, proliferation, inflammation and other processes essential to cancer progression [35]. Increased serum TNFR2 concentrations are associated with type 2 diabetes and acute myocardial infarctions [36, 37]. Previous studies have shown that using antagonistic antibodies to target TNFR2 can suppress the proliferation of ovarian cancer cells and tumour‐associated regulatory T cells [38], highlighting a dually beneficial effect of boosting antitumor immune response and inhibiting tumour cells proliferation. Our finding that inhibiting TNFR2 pharmacologically ameliorates lipolysis suggests a new rationale of targeting TNFR2 against cancer morbidity by attenuating cancer associated cachexia through prevention of adipose wasting and muscle atrophy. Pharmacological inhibition of TNFR2 eliminated the difference in lipolysis between WT and SIRT6 KO adipocytes, suggesting that TNFR2 is involved in the inhibition effects of SIRT6 on lipolysis.

In this study, no change was seen in tumour weight between WT + LLC and TG + LLC mice. This is also explainable. Equal numbers of unmodified LLC cells were subcutaneously injected into both WT and SIRT6 TG mice to form tumours and lead to cachexia. In SIRT6 TG mice, decreased TNFR2 inhibited tumour‐secreted‐TNFα induced adipose lipolysis. However, LLC tumours were not affected by SIRT6 overexpression, and TNFR2 levels were not altered in tumour cells. Therefore, it is not unexpected that tumour weights were similar between WT + LLC and TG + LLC mice. TNFR2 antibody treatment may inhibit both tumour growth and adipose lipolysis in mice, because the antibody can act on tumour cells and adipocytes simultaneously.

SIRT6 functions as an NAD^+^‐dependent histone protein deacetylase with known targets on chromatin being H3K9 and H3K56, resulting in chromatin condensation and gene suppression. We speculate that SIRT6 may function as a deacetylase to suppress TNFR2 expression. In addition, activation of the TNFα receptor has been reported to activate PKA signalling, leading to increased phosphorylation of perilipin 1 [26]. In fact, p‐perilipin was detected by phospho‐PKA substrate antibody in this study [27]. Consistent with this, when SIRT6 was overexpressed, the release of cAMP decreased, but after knockout, the release of cAMP increased (Figure 5D). These data suggested the possible correlation between SIRT6 and PKA signalling pathway in the process of lipolysis inhibition. However, more data are required to prove it.

In our study, overexpression of SIRT6 had no effect on adipocyte differentiation without tumour cell stimulation as shown by Oil red O staining. These data were consistent with the previous report that SIRT6 overexpression does not affect adipogenesis, fatty acid uptake, or lipid oxidation [27]. In fact, the protective effect of SIRT6 overexpression or activation emerges under various of stress. For example, young WT and SIRT6 TG mice exhibit no differences in glucose metabolism; however, aged SIRT6 TG mice (19 months old) show an improved glucose homeostasis compared with their WT counterparts [39]. In addition, while SIRT6 overexpression has no impact on metabolism in mice fed a standard chow diet, it protects against metabolic damage caused by a high‐fat diet and preserves homeostasis in fat and glucose metabolism [13]. We have previously reported that SIRT6 overexpression does not have an obvious effect on mice physically, nevertheless, attenuates cisplatin‐induced renal dysfunction [18]. In conclusion, substantial evidence suggests that SIRT6 is essential throughout life, and elevated SIRT6 levels can mitigate severe diseases. This implies that maintaining or enhancing SIRT6 expression and activity could contribute to counteract various diseases and positively affect health‐span.

MDL800 is a selective SIRT6 activator that can enhance the deacetylase activity of SIRT6 by as much as 22‐fold [29]. It has been shown to have many potentially beneficial applications, including inhibiting the proliferation of human hepatocellular carcinoma (HCC) cells and non‐small cell lung cancer cells, improving the pluripotency of old murine‐derived iPSCs, and alleviating liver fibrosis [40]. Indeed, we observed almost a full reversal of LLC‐induced lipolysis with 20‐μM MDL800, as measured by glycerol release and phosphorylation of perilipin 1 and HSL.

Altogether, our findings join many others in providing a case for SIRT6 activators and agonists as multi‐faceted strategies in the prevention and treatment of cancer cachexia.

## Author Contributions

K.X., K.Y., Z.L. and Z.W. conceived the study and designed the experiments; F.W. and K.Y. collected human sample; K.X., Y.W. and F.W. conducted most experiments and data analyses; Y.G. and Y.R. performed animal feeding, dissection and tissue staining; Y.Q. and X.L. conducted staining analyses and quantification; X.L., Y.Q., Q.L. and Z.W. contributed to the discussion and data interpretation; K.X. and Y.W. drafted the manuscript; V.L., S.C. and all corresponding authors edited the manuscript. All authors reviewed and approved the final manuscript.

## Ethics Statement

All experiments involving animals were carried out in strict adherence to the ethical policies and procedures approved by Institutional Animal Care and Use Committee (IACUC) of Tsinghua University (Approval No. 18‐WZ1). Additionally, written informed consent was secured from each cancer patient and healthy volunteer before collection of blood samples.

## Conflicts of Interest

The authors declare no conflicts of interest.

## Supporting information


**Figure S1.** The expression of SIRT6 in adipose and muscle tissues from WT and SIRT6 TG mice.
**Figure S2.** SIRT6 overexpression prevents muscle atrophy in tumour‐bearing mice.
**Figure S3.** SIRT6 overexpression altered the expression of lipolysis‐related genes.
**Figure S4.** Mature adipocytes differentiated from MEFs were verified.
**Figure S5.** TNFR2 mediates the function of SIRT6 on LLC‐induced lipolysis.
**Figure S6.** The effect of MDL800 on LLC‐induced adipocytes lipolysis.
**Table S1.** Characteristics of study groups.

## Data Availability

All data can be found in the manuscript or the supporting information. Requests for materials or correspondence should be directed to the corresponding author.
