# Supplementary material for: SIRT6 Ameliorates Cancer Cachexia–Associated Adipose Wasting by Suppressing TNFR2 Signalling in Mice
Source: J Cachexia Sarcopenia Muscle. 2025 Feb 19;16(1):e13734. doi: 10.1002/jcsm.13734 (PMC11839279; doi:10.1002/jcsm.13734)
Supplement: Supplementary file 1 — Figure S1. The expression of SIRT6 in adipose and muscle tissues from WT and SIRT6 TG mice. Figure S2. SIRT6 overexpression prevents muscle atrophy in tumour‐bearing mice. Figure S3. SIRT6 overexpression altered the expression of lipolysis‐related genes. Figure S4. Mature adipocytes differentiated from MEFs were verified. Figure S5. TNFR2 mediates the function of SIRT6 on LLC‐induced lipolysis. Figure S6. The effect of MDL800 on LLC‐induced adipocytes lipolysis. Table S1. Characteristics of study groups. [file JCSM-16-e13734-s001.docx]

**Supplementary Figures**

**
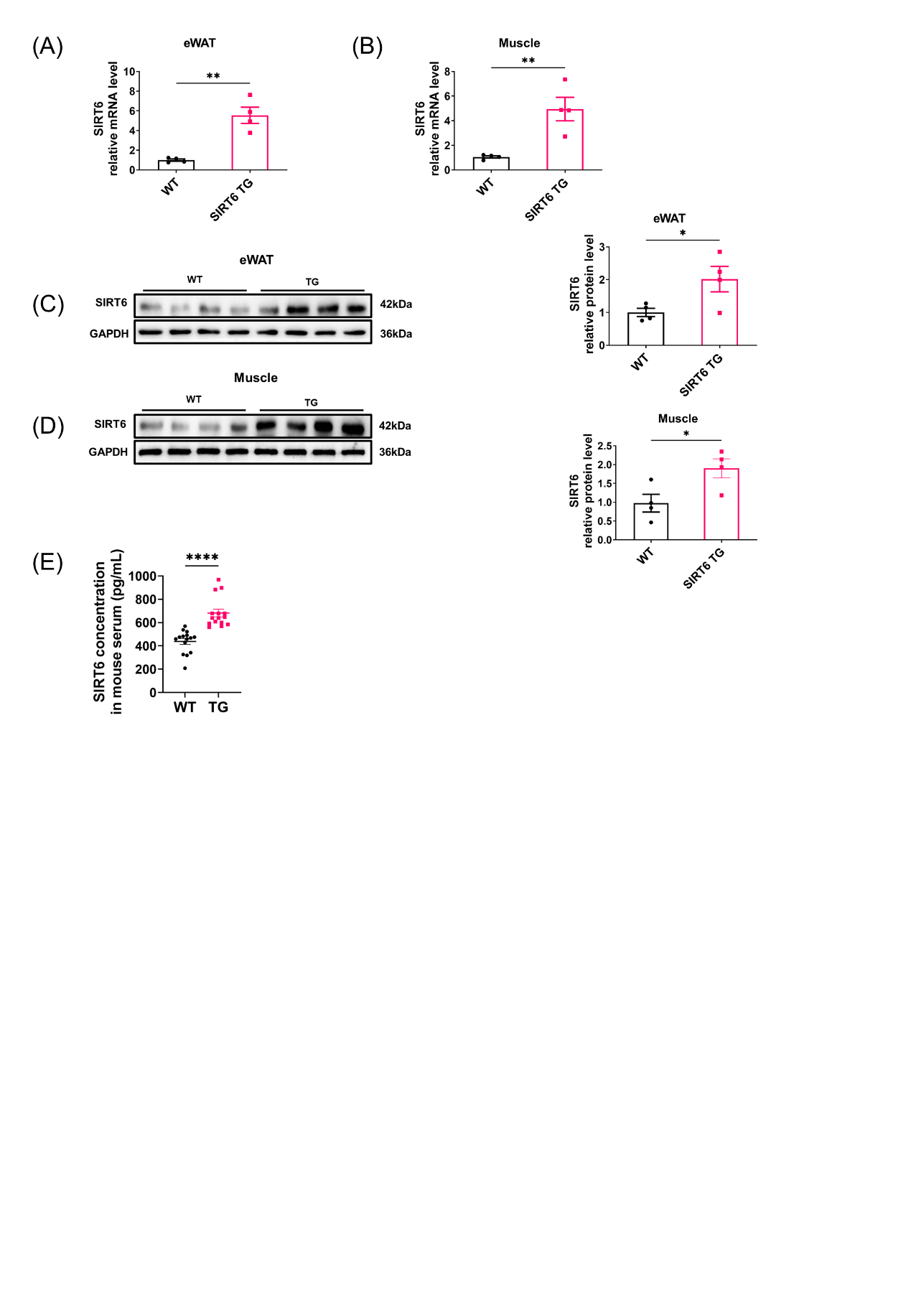
**

**Figure S1. The expression of SIRT6 in adipose and muscle tissues from WT and SIRT6 TG mice.**

(A) The relative mRNA levels of SIRT6 in the eWAT from WT and SIRT6 TG mice were determined by qRT-PCR (n=4 per group).

(B) The relative mRNA levels of SIRT6 in the muscle tissues from WT and SIRT6 TG mice were determined by qRT-PCR (n=4 per group).

(C) The relative protein levels of SIRT6 in the eWAT from WT and SIRT6 TG mice were determined by western blot (n=4 per group).

(D) The relative protein levels of SIRT6 in the muscle tissues from WT and SIRT6 TG mice were determined by western blot (n=4 per group).

**(E) Serum SIRT6 concentrations in 10-week-old male WT mice (n=15) and SIRT6 TG mice (n=15) were compared.**

^*^*p*<0.05, ^**^*p*<0.01**, ^****^*p*<0.0001**

**
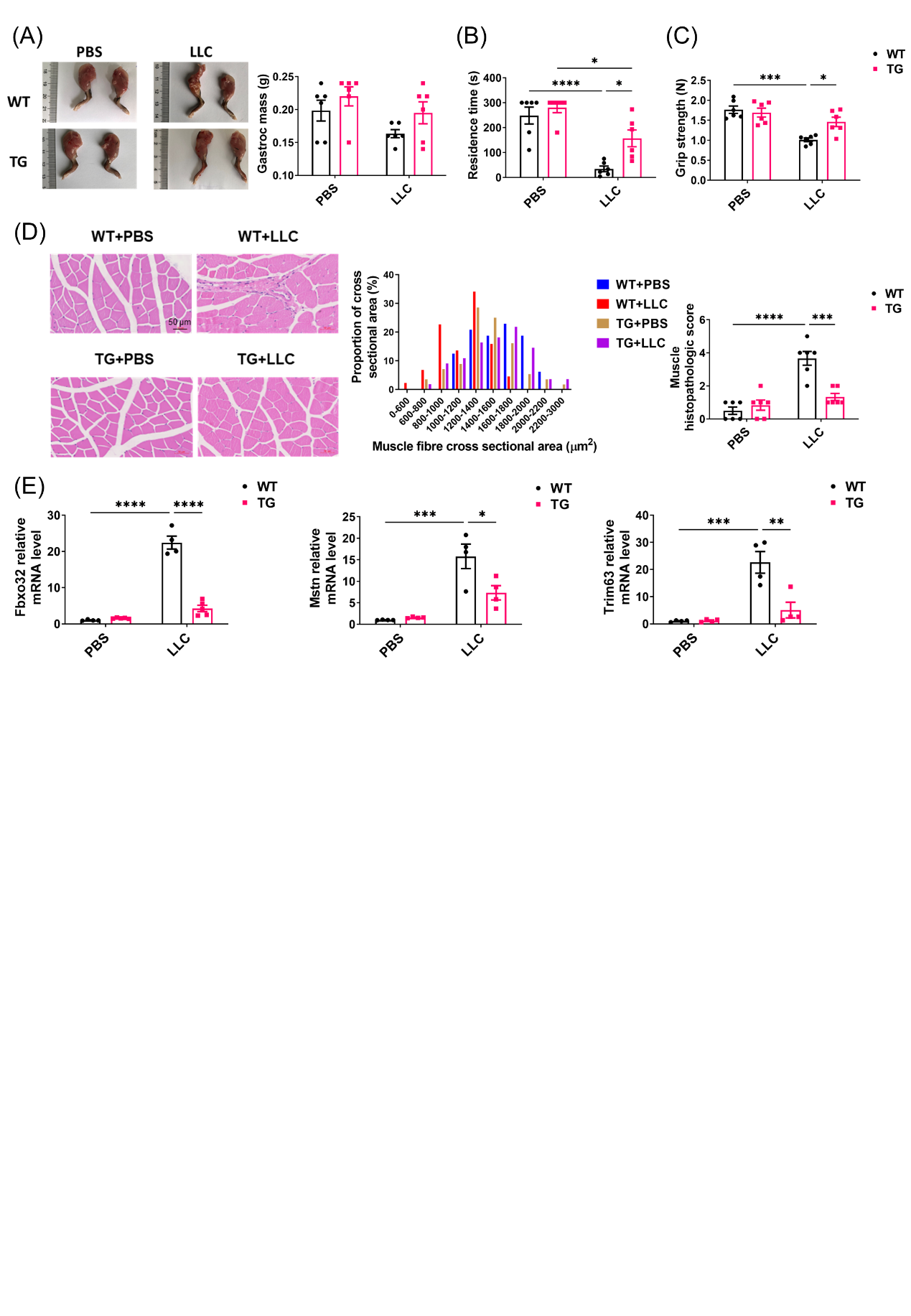
**

**Figure S2. SIRT6 overexpression prevents muscle atrophy in tumor-bearing mice.**

(A) SIRT6 transgenic (TG) and wild type (WT) mice were inoculated with LLC cells or PBS and euthanized 21 days after tumor injection. Representative images of the hindlimb and weight of the gastrocnemius muscle (Gastroc) mass were shown.

(B-C) Muscle function was analyzed by measuring residence time in the rotarod test (B) and grip strength (C) (n=6 per group).

(D) Representative H&E-stained images of muscle section. Size distribution and histopathological score of muscle myofibers were quantified (n=6 per group).

(E) The mRNA levels of muscle atrophy markers in the gastroc were determined by quantitative reverse transcription PCR (qRT-PCR) (n=4 per group).

**p*<0.05, ***p*<0.01, ****p*<0.001, *****p*<0.0001

**
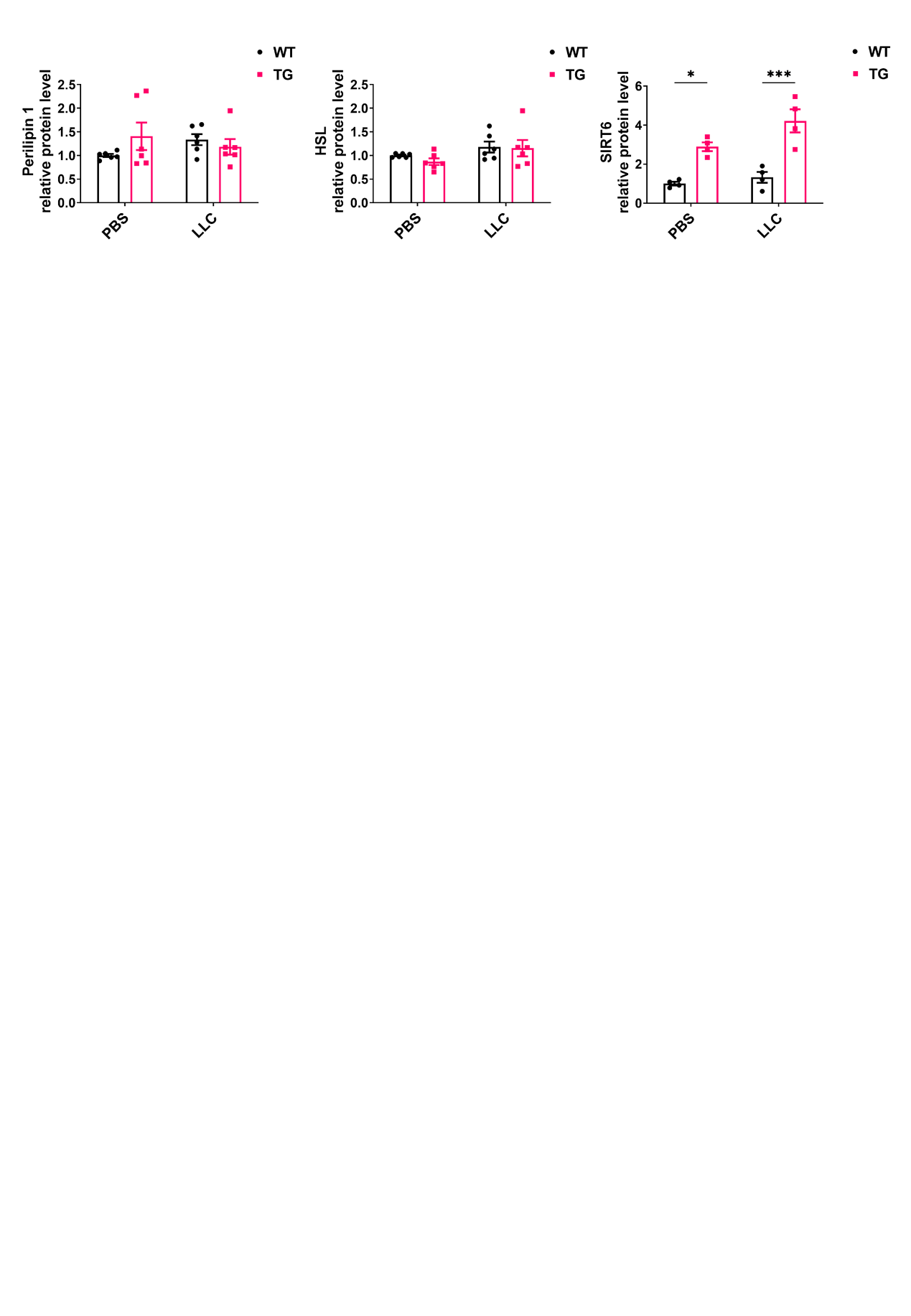
**

**Figure S3. SIRT6 overexpression altered the expression of lipolysis-related genes.**

Quantification of western blot in Figure 3C (n=6 per group).

^*^*p*<0.05, ^***^*p*<0.001


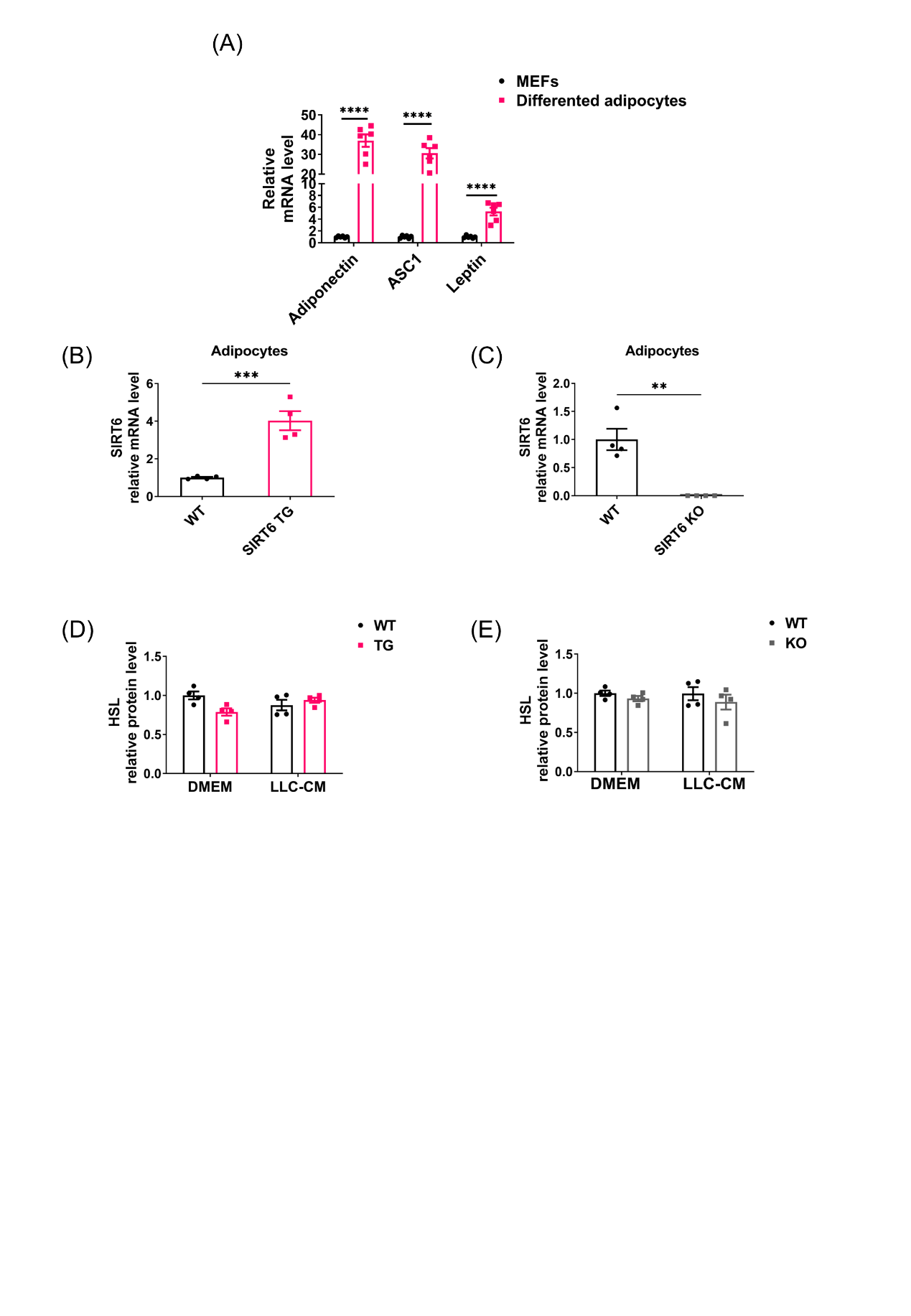


**Figure S4. Mature adipocytes differentiated from MEFs were verified.**

1. The mRNA levels of Adiponectin, ASC1 and Leptin in MEFs and differentiated adipocytes (n=6 per group).

**(B-C) SIRT6 expression in SIRT6 TG adipocytes (B) and SIRT6 KO adipocytes (C) were measured (n=4 per group).**

**(D-E) The expression of HSL in different adipocytes were determined by western blot (n=4 per group).**

*****p*<0.01, ****p*<0.001,** ^****^*p*<0.0001


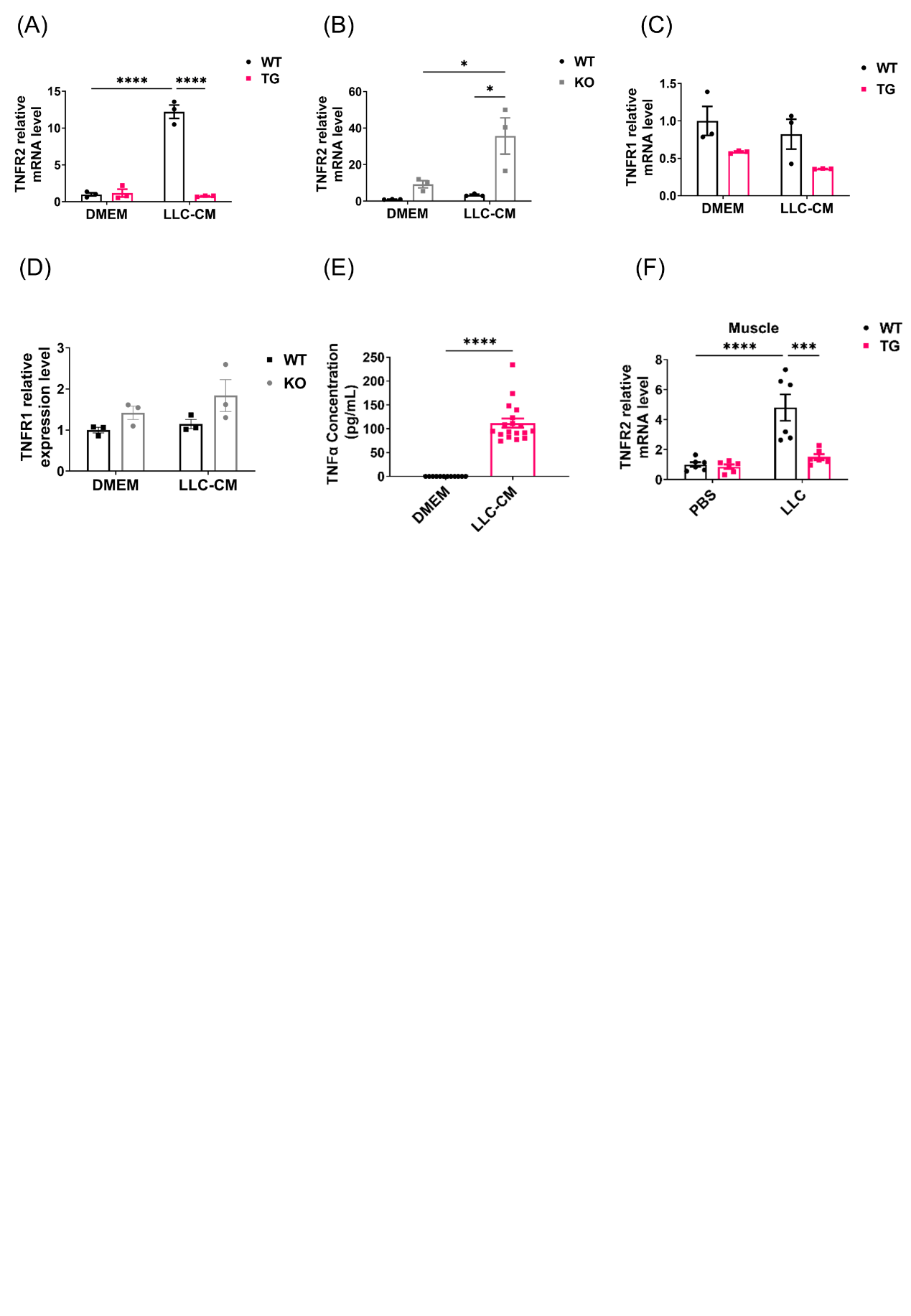


**Figure S5. TNFR2 mediates the function of SIRT6 on LLC-induced lipolysis.**

(A) TNFR2 mRNA levels in WT+DMEM (WT adipocytes were treated with DMEM medium), WT+LLC (WT adipocytes were treated with LLC cell-conditioned medium), TG+DMEM (SIRT6 TG adipocytes were treated with DMEM medium), TG+LLC (SIRT6 TG adipocytes were treated with LLC cell-conditioned medium) were determined by qRT-PCR (n=3 per group).

(B) TNFR2 mRNA levels in WT+DMEM (WT adipocytes were treated with DMEM medium), WT+LLC (WT adipocytes were treated with LLC cell-conditioned medium), KO+DMEM (SIRT6 KO adipocytes were treated with DMEM medium), KO+LLC (SIRT6 KO adipocytes were treated with LLC cell-conditioned medium) were determined by qRT-PCR (n=3 per group).

(C) TNFR1 mRNA levels in WT+DMEM, WT+LLC, TG+DMEM, TG+LLC were determined by qRT-PCR (n=3 per group).

(D) TNFR1 mRNA levels in WT+DMEM, WT+LLC, KO+DMEM, KO+LLC were determined by qRT-PCR (n=3 per group).

(E) TNFα concentrations of DMEM medium (n=12) and LLC cell-conditioned medium (n=18) were compared.

(F) TNFR2 mRNA levels in muscle tissues from WT+PBS, WT+LLC, SIRT6 TG+PBS, SIRT6 TG+LLC mice were determined by qRT-PCR (n=6 per group).

^*^*p*<0.05, ^***^*p*<0.001, ^****^*p*<0.0001

**
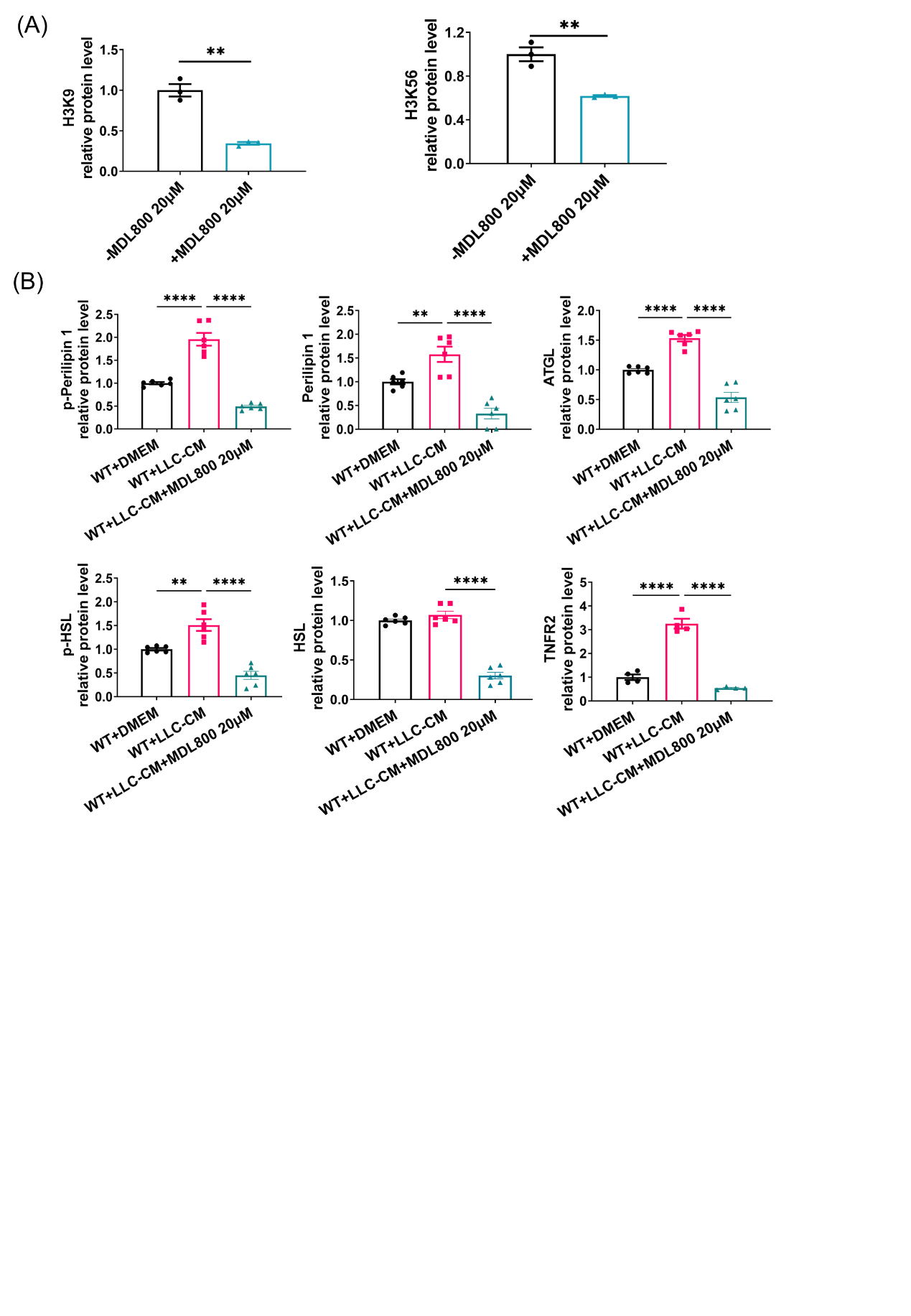
**

**Figure S6. The effect of MDL800 on LLC-induced adipocytes lipolysis.**

Quantification of western blot in Figure 6A (A, n=3 per group) and Figure 6D (B, n=6 per group; TNFR2, n=4 per group).

^**^*p*<0.01, ^****^*p*<0.0001

**Supplementary Tables**


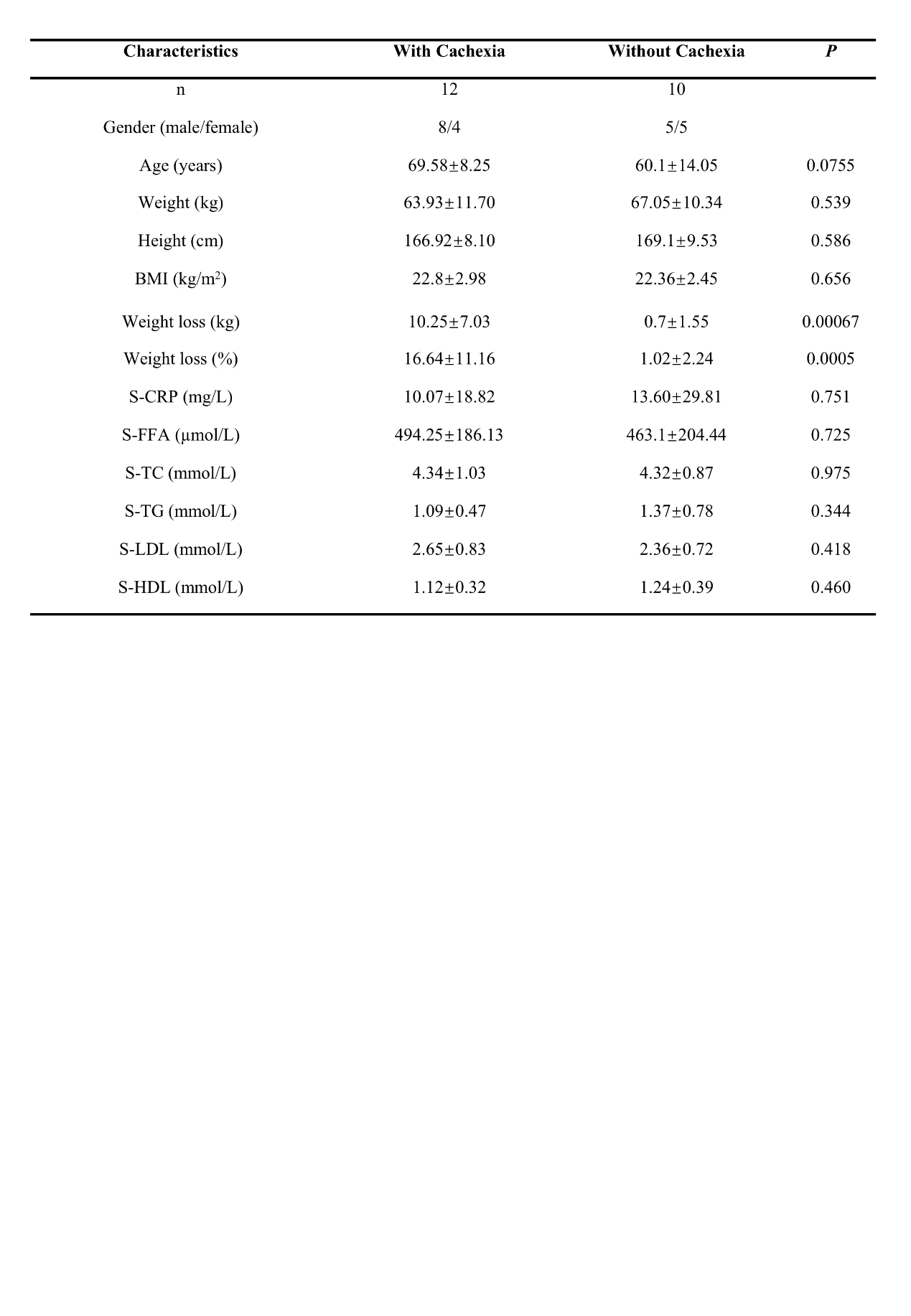


**Table S1** Characteristics of study groups

BMI, body mass index; S, serum; CRP, C-reactive protein; FFA, free fatty acid; TC, total cholesterol; TG, triglyceride; LDL, low-density lipoprotein; HDL, high-density lipoprotein. Values are mean ± standard deviation.
